# Recurrences after *nephron-sparing* treatments of renal cell carcinoma: a competing risk analysis

**DOI:** 10.1007/s00345-024-05172-1

**Published:** 2024-08-07

**Authors:** Andreas Karlsson Rosenblad, Bassam Mazin Hashim, Per Lindblad, Börje Ljungberg, the National Swedish Kidney Cancer Register Steering Committee

**Affiliations:** 1 Regional Cancer Centre Stockholm-Gotland, Region Stockholm, Stockholm, Sweden; 2 Department of Statistics, Uppsala University, Uppsala, Sweden; 3 Department of Medical Sciences, Division of Clinical Diabetology and Metabolism, Uppsala University, Uppsala, Sweden; 4 Department of Neurobiology, Care Sciences and Society, Division of Family Medicine and Primary Care, Karolinska Institutet, Stockholm, Sweden; 5 Department of Surgical Sciences, Center for Clinical Research, County of Västmanland, Uppsala University, Uppsala, Sweden; 6 Faculty of Medicine and Health, School of Medical Sciences, Örebro University, Örebro, Sweden; 7 Department of Diagnostics and Intervention, Urology and Andrology, Umeå University, Umeå, Sweden; 8 Department of Diagnostics and Intervention, Urology and Andrology, Umeå University, 901 85 Umeå, Sweden

**Keywords:** Ablative therapy, Distant metastatic recurrence, Kidney cancer, Local recurrence, Renal cell carcinoma, Partial nephrectomy

## Abstract

**Purpose:**

To examine associations between ablative therapy (AT) and partial nephrectomy (PN) and the occurrence of local recurrence (LR), distant metastatic recurrence (DMR) and all-cause mortality in a nation-wide real-world population-based cohort of patients with nonmetastatic renal cell carcinoma (nmRCC).

**Methods:**

Data on 2751 AT- or PN-treated nmRCC tumours diagnosed during 2005–2018, representing 2701 unique patients, were obtained from the National Swedish Kidney Cancer Register. Time to LR/DMR or death with/without LR/DMR was analysed using Cox regression models.

**Results:**

During a mean of 4.8 years follow-up, LR was observed for 111 (4.0%) tumours, DMR for 108 (3.9%) tumours, and death without LR/DMR for 206 (7.5%) tumours. AT-treated tumours had a 4.31 times higher risk of LR (P < 0.001) and a 1.91 times higher risk of DMR (P = 0.018) than PN-treated, with no significant differences in risk of death without LR/DMR. During a mean of 3.2 and 2.5 years of follow-up after LR/DMR, respectively, 24 (21.6%) of the LR cases and 56 (51.9%) of the DMR cases died, compared to 7.5% in patients without LR/DMR. There were no significant differences between AT- and PN-treated regarding risks of early death after occurrence of LR or DMR.

**Conclusion:**

AT treatment of patients with nmRCC implied significantly higher risks of LR and DMR compared with PN treatment. To minimize the risks of LR and DMR, these results suggest that PN is preferred over AT as primary treatment, supporting the EAU guidelines to recommended AT mainly to frail and/or comorbid patients.

## Introduction

Partial nephrectomy (PN) or ablative therapy (AT) with a curative intent for patients with localized nonmetastatic renal cell carcinoma (nmRCC) imply risks for local recurrence (LR) and distant metastatic recurrence (DMR) [1]. The risk for LR affecting the tumour-bearing kidney increases with tumour size, multifocality, incomplete treatment, and vascular and surrounding tissue invasion at the time of treatment [2, 3].

Following AT, LR have been reported in 14% of the cases [4]. Whereas repeated ablation is recommended as the preferred option after AT failure, the most effective salvage procedure is not defined [5]. LR after PN occurs in about 3% of the cases, more commonly in larger tumours and higher stages [1]. Despite limited evidence, surgical removal of locally recurrent disease can induce durable tumour control if negative margins are achieved, although with risks of complications [6].

Since LR develops within 20–36 months after treatment [7], surgical or AT treatment of LR in absence of systemic progression may be considered. However, the clinical effectiveness of AT compared with PN for nmRCC patients, in terms of occurrence of LR/DMR and all-cause mortality (ACM), has not been settled.

### Aim

To examine the association between AT/PN treatment and occurrence of LR/DMR and ACM in a population-based cohort of nmRCC patients.

## Material and methods

### Study design and participants

**Fig. 1:**
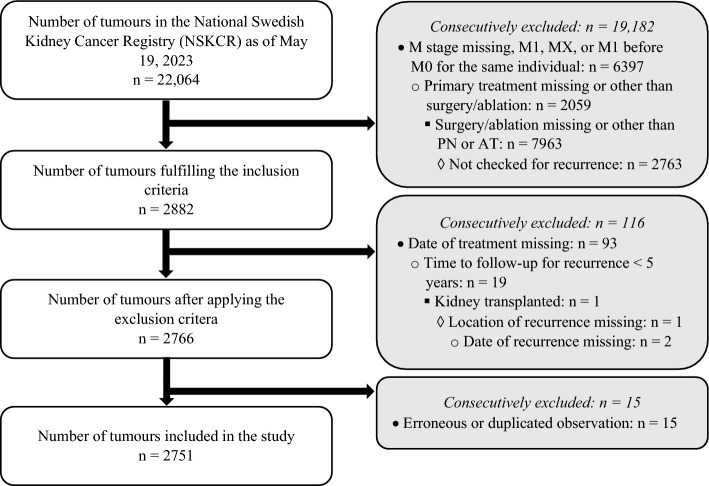
Flowchart of the inclusion process for the present study

This study included metastasis-free patients registered in the National Swedish Kidney Cancer Register (NSKCR) [8–13] from 2005 to May 19, 2023 with AT or PN as primary treatment who had been followed-up for recurrences. Exclusion criteria included missing date of treatment or date/location of recurrence, follow-up time for recurrence < 5 years, and transplanted kidney. After applying these criteria, 2751 tumours remained, representing 2701 unique individuals, of which 47 (1.7%) had more than one tumour (Fig. 1). Reasons for being included more than once in the NSKCR are having bilateral RCCs treated at different dates or multifocal tumours with different morphologies.

### Data collection

The TNM 2017 classification system [14] was used for tumour staging, with tumour size defined as the maximal tumour diameter obtained using computed tomography or magnetic resonance imaging. Histopathological classification was performed according to the 2016 WHO classification [15]. Date of first observed LR/DMR was obtained by retrospectively examining each patient’s medical records at 5 and 10 years after RCC diagnosis, while dates of death/emigration were obtained from the Swedish Population Register. Individuals experiencing LR and DMR at the same date were categorised as DMR.

Data on treatment, follow-up time for recurrence, type/site of recurrence, TNM stage, tumour size, RCC type, incidental finding, kidney transplantation and dates of diagnosis, treatment, and recurrence were retrieved from the NSKCR. Sex and date of birth were obtained from the patient’s Swedish Personal Identification Number.

### Statistical analyses

Categorical data are presented as frequencies and percentages, n (%), while continuous data are given as mean values with standard deviations (SDs). Hypotheses of differences between AT- and PN-treated were tested using generalized estimated equations (GEE) models with AT (yes/no) as explanatory variable. The GEE models used unstructured correlation structures and applied a Gaussian error distribution with identity link for continuous outcomes and a binomial error distribution with logit link for binary outcomes [16]. For multinomial outcomes, the local odds ratios GEE approach for nominal responses was applied, using a time exchangeability structure and a baseline category logit link [17].

To estimate the association between AT/PN treatment and occurrence of LR/DMR/ACM, competing risks Cox regression models were used with the outcome time (years) from date of treatment to date of first event (LR, DMR, or death without LR/DMR) or censoring (emigrated or alive at follow-up without having experienced LR/DMR). For individuals experiencing LR/DMR, standard Cox regression models were used with the outcome time (years) from date of LR/DMR to date of death or censoring (emigrated or alive at follow-up). To avoid possible biases due to differences in chances of having recurrences recorded, each individual was only followed-up until 5 or 10 years after the date of RCC diagnosis, whichever came later, provided that this occurred on or before May 19, 2023. Robust variances were used for all models, with clustering of observations for the same individual.

Adjusted regression models used age at treatment (years), waiting time from diagnosis to treatment (years), calendar year of treatment (2005–2022), male sex, tumour size (mm), T stage (T1a [reference category]/T1b/T2–T4), and RCC type (clear cell [ccRCC; reference category], papillary/chromophobe, or other) as confounders. Incidental finding was excluded due to having > 1% missing values. Results are presented as hazard ratios (HRs) with 95% confidence intervals (CIs). All statistical analyses were performed in R 4.3.1/4.4.1 (R Foundation for Statistical Computing, Vienna, Austria), with two-sided P-values < 0.05 considered statistically significant.

### Ethical approval

The study was approved by the Ethical Review Board of Northern Sweden (Dnr: 2012-418-31 M) and the Swedish Ethical Review Agency (Dnr: 2019-2579 and 2020-05093).

## Results

**Fig. 2:**
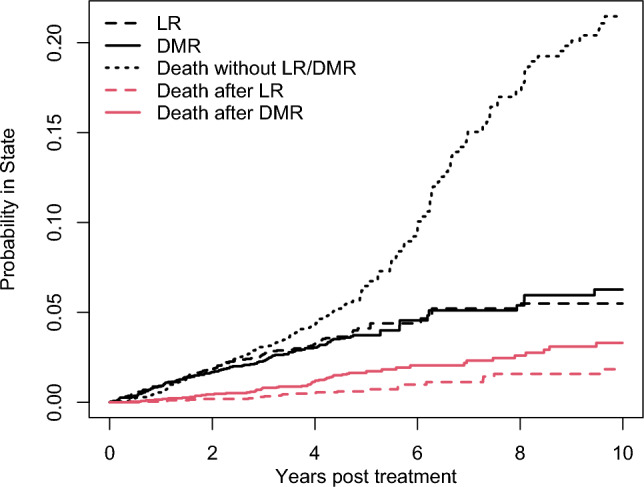
Probability of being in any of the five possible states (LR, DMR, death without LR/DMR, death after LR, and death after DMR) during the ten years post treatment. Notably, the LR/DMR curves include those who have died after LR/DMR, and the proportion of subjects being in the LR/DMR state at any given time is thus given by the distance between the LR/DMR curves and the curves for those who have died after LR/DMR

Table 1 gives the characteristics of the 2751 nmRCCs included in this study. After 13,299 person-years (mean [SD] 4.8 [1.8] years) of follow-up from treatment to first event/censoring, LR was observed for 111 (4.0%) tumours, DMR for 108 (3.9%) tumours, and death without LR/DMR for 206 (7.5%) tumours. LR/DMR events were followed-up until death/censoring during 351 and 268 person-years (mean [SD] 3.2 [2.2] and 2.5 [2.3] years), respectively, with 24 (21.6%) LR and 56 (51.9%) DMR cases dying. Figure 2 gives the ten-years probability of being in any of the five possible states (LR, DMR, death without LR/DMR, death after LR, and death after DMR).

**Table 1:** Characteristics of the n = 2751 tumours (representing 2701 unique individuals) treated with ablation therapy (AT) or partial nephrectomy (PN) included in the study

Variable	All n = 2751 (100.0%)	Treatment		P-value
		AT^a^n = 453 (16.5%)	PN^b^n = 2298 (83.5%)	
Time to follow-up (years),^c^ median; mean (SD)				N/A
– Time from treatment to first event^d^ or censoring	4.7; 4.8 (1.8)	4.5; 4.2 (1.7)	4.8; 5.0 (1.8)	
– Time from LR to death or censoring	2.9; 3.2 (2.2)	3.3; 3.6 (2.4)	2.7; 2.9 (2.1)	
– Time from DMR to death or censoring	1.8; 2.5 (2.3)	1.2; 2.1 (2.3)	1.8; 2.6 (2.3)	
Status at follow-up, n (%)				N/A
– Censored w/o LR or DMR	2326 (84.6)	335 (74.0)	1991 (86.6)	
– Deceased w/o LR or DMR	206 (7.5)	50 (11.0)	156 (6.8)	
– Local recurrence (LR)	111 (4.0)	44 (9.7)	67 (2.9)	
• Deceased after LR	24 (21.6)	7 (15.9)	17 (25.4)	
– Distant metastatic recurrence (DMR)^e^	108 (3.9)	24 (5.3)	84 (3.7)	
• Deceased after DMR	56 (51.9)	14 (58.3)	42 (50.0)	
Age at treatment (years), mean (SD)	64.0 (11.8)	69.3 (11.1)	63.0 (11.6)	** < 0.001**^ h^
Waiting time from diagnosis to treatment (days), mean (SD)	139 (152)	208 (206)	125 (135)	** < 0.001**^ h^
Male sex, n (%)	1814 (65.9)	286 (63.1)	1528 (66.5)	0.191^i^
Incidental finding, n (%)^f^	2152 (81.0)	374 (87.2)	1778 (79.8)	**0.004**^i^
Tumour size (mm), mean (SD)	32.6 (16.4)	27.5 (12.7)	33.7 (16.8)	** < 0.001**^ h^
T stage, n (%)^g^				
– T1a	2117 (77.3)	410 (91.5)	1707 (74.5)	Ref
– T1b	448 (19.5)	26 (5.8)	448 (19.5)	**0.001**^j^
– T2–T4	149 (5.4)	12 (2.7)	137 (6.0)	0.251^j^
RCC type, n (%)^g^				
– Clear cell	1869 (68.2)	283 (63.7)	1586 (69.1)	Ref
– Papillary/Chromophobe	790 (28.8)	143 (32.2)	647 (28.2)	0.082^j^
– Other	81 (3.0)	18 (4.1)	63 (2.7)	0.355^j^

*Notes:* *AT* ablation therapy, *DMR* distant metastatic recurrence, *LR* local recurrence, *PN* partial nephrectomy, *Ref.* reference category, *SD* standard deviation. Significant P-values are given in **bold**. Type of surgery: ^a^42 (9.4%) Laparoscopic, 407 (90.6%) Percutaneous; Missing values: n = 4 (0.9%); ^b^241 (10.5%) Laparoscopic, 527 (22.9%) Robot-assisted, 1530 (66.6%) Open surgery. ^c^Person-years of follow-up: from treatment 13,299 years, from LR 351 years, from DMR 268 years. ^d^LR, DMR, or death w/o LR or DMR. ^e^4 (0.9%) in the AT group and 14 (0.6%) in the PN group who experienced both LR and DMR were included in the DMR group. Missing values: ^f^n = 93 (3.4%); ^g^n = 11 (0.4%). P-values from GEE models with ^h^Gaussian error distribution and identity link; ^i^binomial error distribution and logit link; ^j^local odds ratios for nominal responses with baseline category logit link

AT-treated were on average older than PN-treated, mean (SD) age 69.3 (11.1) compared to 63.0 (11.6) years (P < 0.001), and waited longer for treatment, mean (SD) 208 (206) compared to 125 (135) days (P < 0.001). Likewise, incidental finding was more common among AT-treated (n = 374; 87.2%) than PN-treated (n = 1778; 79.8%) (P = 0.004), while the mean (SD) tumour size was smaller, 27.5 (12.7) compared to 33.7 (16.8) mm (P < 0.001). Male sex was most common (65.9%), with no significant differences between AT- and PN-treated.

T1a was more common among AT-treated (n = 410; 91.5%) than PN-treated (n = 1707; 74.5%), while T1b was less common among AT-treated (n = 26; 5.8%) than PN-treated (n = 448; 19.5%). While the risk of having T1b tumours was significantly higher among PN-treated (P = 0.001), there were no significant differences in risks of having T2–T4 tumours (P = 0.251). The most common RCC type was ccRCC (68.2%), with no significant differences between AT- and PN-treated.

### Time from treatment to first event and death after LR/DMR

Table 2 gives the results from the Cox regression analyses with the outcomes time from treatment to first event/censoring and time from LR/DMR to death/censoring. In the adjusted analyses, AT-treated had a 4.31 times higher risk of LR (P < 0.001) and a 1.91 times higher risk of DMR (P = 0.018). Of the confounders, only RCC type was significantly associated with risk of LR in the adjusted analyses, with papillary/chromophobe RCCs having a lower risk of LR than ccRCCs, HR 0.53 (P = 0.012).

**Table 2:** Results from adjusted and unadjusted Cox regression models for a) time from treatment with ablation therapy (AT) or partial nephrectomy (PN; reference category) to first event (local recurrence [LR], distant metastatic recurrence [DMR], or death w/o LR or DMR), and b) time from LR/DMR to death

	Variable	Unadjusted			Adjusted^a^		
		HR	95% CI	P-value	HR	95% CI	P-value
*Event*	*(a) Time from treatment to first event*						
LR	Ablation therapy	3.83	2.60 –5.62	** < 0.001**	4.31	2.79–6.66	** < 0.001**
	Age at treatment (years)	1.02	0.99 –1.04	0.089	1.00	0.98–1.02	1.000
	Waiting time from diagnosis to treatment (years)	1.38	0.95 –2.01	**0.084**	1.19	0.74–1.91	0.470
	Calendar year of treatment	1.03	0.94 –1.13	0.469	1.02	0.93–1.12	0.690
	Male sex	0.86	0.58 –1.27	0.445	0.91	0.61–1.34	0.618
	Tumour size (mm)	1.01	0.99–1.02	0.179	1.01	0.99–1.02	0.300
	T stage						
	– T1a	Ref			Ref		
	– T1b	1.31	0.82–2.08	0.254	1.47	0.83–2.58	0.177
	– T2–T4	1.11	0.48–2.56	0.809	1.13	0.33–3.83	0.848
	RCC type						
	– Clear cell	Ref			Ref		
	– Papillary/Chromophobe	0.55	0.33–0.90	**0.016**	0.53	0.32–0.87	**0.012**
	– Other	1.10	0.40–2.99	0.859	0.93	0.33–2.58	0.888
DMR	Ablation therapy	1.67	1.06–2.63	**0.025**	1.91	1.11–3.28	**0.018**
	Age at treatment (years)	1.05	1.02–1.07	** < 0.001**	1.04	1.01–1.07	** < 0.001**
	Waiting time from diagnosis to treatment (years)	0.92	0.47–1.76	0.799	0.91	0.46–1.79	0.780
	Calendar year of treatment	0.89	0.82–0.97	**0.004**	0.87	0.79–0.94	** < 0.001**
	Male sex	1.49	0.97–2.31	0.068	1.63	1.06–2.52	**0.026**
	Tumour size (mm)	1.02	1.01–1.03	** < 0.001**	1.01	1.00–1.03	**0.015**
	T stage						
	– T1a	Ref			Ref		
	– T1b	2.40	1.55–3.71	** < 0.001**	1.85	1.11–3.08	**0.018**
	– T2–T4	4.26	2.48–7.31	** < 0.001**	2.71	1.25–5.88	**0.011**
	RCC type						
	– Clear cell	Ref			Ref		
	– Papillary/Chromophobe	0.60	0.37–0.97	**0.036**	0.55	0.34–0.90	**0.016**
	– Other	0.85	0.26–2.73	0.788	0.75	0.21–2.62	0.651
Death w/o LR or DMR	Ablation therapy	2.13	1.54–2.93	** < 0.001**	1.35	0.95–1.91	0.094
	Age at treatment (years)	1.09	1.07–1.12	** < 0.001**	1.08	1.06–1.11	** < 0.001**
	Waiting time from diagnosis to treatment (years)	1.77	1.39–2.26	** < 0.001**	1.52	1.15–2.01	**0.003**
	Calendar year of treatment	1.18	1.07–1.31	** < 0.001**	1.13	1.03–1.24	**0.008**
	Male sex	1.04	0.78–1.40	0.769	1.07	0.79–1.43	0.662
	Tumour size (mm)	1.01	1.00–1.02	** < 0.001**	1.02	1.00–1.03	** < 0.001**
	T stage						
	– T1a	Ref			Ref		
	– T1b	1.35	0.95–1.91	0.084	0.83	0.54–1.27	0.396
	– T2–T4	1.10	0.59–2.03	0.756	0.45	0.17–1.18	0.104
	RCC type						
	– Clear cell	Ref			Ref		
	– Papillary/Chromophobe	1.07	0.79–1.45	0.668	1.00	0.73–1.36	0.976
	– Other	1.06	0.47–2.40	0.884	1.03	0.46–2.30	0.946
*Recurrence*	*(b) Time from LR/DMR to death*						
LR	Ablation therapy	0.50	0.21–1.19	0.115	0.49	0.18–1.29	0.148
	Age at treatment (years)	1.02	0.99–1.05	0.147	1.02	0.99–1.05	0.220
	Waiting time from diagnosis to treatment (years)	0.56	0.21–1.50	0.247	0.72	0.21–2.40	0.596
	Calendar year of treatment	1.15	0.93–1.41	0.187	1.23	0.98–1.56	0.072
	Male sex	0.66	0.30–1.44	0.295	0.52	0.20–1.33	0.168
	Tumour size (mm)	1.03	1.00–1.05	**0.028**	1.05	0.98–1.12	0.165
	T stage						
	T1a	Ref			Ref		
	T1b	1.32	0.55–3.17	0.535	0.39	0.05–2.66	0.335
	T2–T4	2.84	0.69–11.50	0.144	1.48	0.11–18.98	0.761
	RCC type						
	Clear cell	Ref			Ref		
	Papillary/Chromophobe	0.38	0.10–1.38	0.141	0.30	0.06–1.44	0.131
	Other	2.92	0.90–9.43	0.074	1.32	0.23–7.49	0.752
DMR	Ablation therapy	1.36	0.69–2.66	0.373	1.26	0.58–2.73	0.557
	Age at treatment (years)	1.00	0.97–1.03	0.839	1.00	0.96–1.03	0.815
	Waiting time from diagnosis to treatment (years)	1.39	0.57–3.34	0.462	1.31	0.51–3.36	0.568
	Calendar year of treatment	1.06	0.94–1.21	0.317	1.06	0.92–1.21	0.409
	Male sex	1.37	0.72–2.59	0.329	1.16	0.58–2.31	0.673
	Tumour size (mm)	1.01	0.99–1.02	0.410	1.00	0.97–1.03	0.970
	– T stage						
	– T1a	Ref			Ref		
	– T1b	1.49	0.82–2.67	0.183	1.64	0.65–4.11	0.289
	– T2–T4	1.47	0.67–3.22	0.336	1.50	0.48–4.64	0.479
	– RCC type						
	– Clear cell	Ref			Ref		
	– Papillary/Chromophobe	1.25	0.70–2.23	0.443	1.10	0.54–2.22	0.786
	– Other	0.51	0.05–4.42	0.544	0.40	0.01–8.37	0.551

*Notes:* *CI* confidence interval, *DMR* distant metastatic recurrence, *HR* hazard ratio, *LR* local recurrence, *Ref.* reference category, *SE* standard error. Significant P-values are given in **bold**. ^a^Results for time from treatment to first event based on 2730 (99.2%) observations with 422 (15.5%) events (109 LR, 107 DMR, 206 death w/o LE or DMR); concordance (SE) 0.712 (0.013). Results for time from LR to death based on 109 (98.2%) observations with 24 (22.0%) events; concordance (SE) 0.709 (0.054). Results for time from DMR to death based on 107 (99.1%) observations with 55 (51.4%) events; concordance (SE) 0.601 (0.045)

Being one year older implied 1.04 times higher DMR risk (P < 0.001), being male a 1.63 times higher risk (P = 0.026) and having a one mm larger tumour a 1.01 times higher risk (P = 0.015). Having a T1b tumour entailed 1.85 times higher DMR risk compared with having a T1a tumour (P = 0.018), while having a T2–T4 tumour implied a 2.71 times higher risk (P = 0.011). More recently treated tumours had a significantly lower DMR risk, HR 0.87 for each additional calendar year (P < 0.001), while papillary/chromophobe RCCs had a significantly lower DMR risk than ccRCCs, HR 0.55 (P = 0.016).

AT-treated had a significantly higher risk of death without LR/DMR in the unadjusted analyses, HR 2.13 (P < 0.001), but not in the adjusted analyses, HR 1.35 (P = 0.094). However, age, waiting time, year of treatment, and tumour size were all significant in the adjusted analyses. Being one year older implied a 1.08 times higher risk for death without LR/DMR (P < 0.001), waiting one additional year for treatment a 1.52 times higher risk (P = 0.003), having the treatment performed one calendar year later a 1.13 times higher risk (P = 0.008), and having a one mm larger tumour a 1.02 times higher risk (P < 0.001).

In the unadjusted analyses, a one mm larger tumour implied a 1.03 times higher risk of early death after LR (P = 0.028). However, this significant association disappeared in the adjusted analyses (P = 0.165). No other variable was significantly associated with risk of early death after having LR/DMR in adjusted or unadjusted analyses.

## Discussion

This study on AT- or PN-treated nmRCC tumours showed that AT treatment entailed significantly increased risks of LR and DMR. However, there were no significant differences between AT- and PN-treated regarding risks of early death.

### Results in perspective

The increased use of minimal invasive treatments such as AT has been encouraged by reported clinical efficiency of AT over PN and lower costs [18, 19]. However, comparative data are severely biased with marked patients’ selection and sometimes also interspersion with benign histology [20–23]. Most studies comparing AT and PN treatments have been retrospective studies with poorly matched controls and short follow-up[20]. A systematic review found that AT had few complications, while its long-term oncological effectiveness compared with PN remained uncertain [20]. A recent multicenter study comparing AT- and PN-treated cT1a-RCC patients found similar 90 days quality-of-life impacts [24]. Thus, the optimal treatment for small RCCs has not been settled. This study showed a nearly twofold higher risk for LR and fourfold higher risk for DMR for AT-treated compared with PN-treated, although there were no significant differences in risks of early death. The mean time to the occurrence of LR and DMR might seems to reflect the biology of the disease being in the best prognostic stage group. Comparable results on 5-year cancer-specific survival has been observed in the SEER database comparing AT and PN treatment [25]. LR was observed in 9.5% of AT-treated compared to 2.9% of PN-treated patients. These results are in line with results from the Canadian Kidney Cancer Information system comprising 2276 patients [26], which used treatment modality (PN versus AT) as a predictor of disease recurrence. A systematic review and meta-analysis comprising 27 studies and 13,996 patients found that PN was associated with a lower LR risk than AT, but found no significant difference in DMR risk [27]. For T1b-RCC patients, a multicentre comparative matched-pairs analysis of percutaneous AT and robot assisted PN found significantly higher LR rates in the AT (14.6%) than the PN (4.0%) group [28]. In contrast to our findings, this latter study found no significant difference in occurrence of DMR [21]. For patients with solitary kidneys, AT had higher recurrence rate (29%) than PN (3.2%) and worse recurrence-free survival (P = 0.027), although PN was afflicted with more common acute kidney injury postoperatively while eGFR was similar at 3 months follow-up [29]. The reason for higher proportions of local and distant recurrence in the present study cannot be determined but might be due to all kinds of technical differences between AT and PN.

Although AT was associated with good long-term survival in several single-arm and retrospective comparative studies [23, 24], this apparent benefit is subject to considerable uncertainties due to poorly matched controls and studies with low-quality evidence [17]. The EAU guidelines concluded that AT is recommended only to frail and/or comorbid patients with small tumours [5]. Our study supports this recommendation, showing a lower risk of LR and DMR for PN than AT treatment.

### Strengths and limitations

A strength of this study was the use of high-quality validated real-world data [30] from a nation-wide population-based register, having a large sample size of 2751 non-metastatic RCCs with a total of 13,299 person-years of follow-up to first event. Another strength was the adjustment for year of treatment in the statistical analyses, aiming to balance learning curve improvements. An important limitation was the lack of control for comorbidities in the statistical analyses: potentially influencing the association between treatment and outcome Other limitations were the relatively small sample of patients experiencing LR/DMR, making these analyses under-powered. Furthermore, type of ablation was not fully available. Finally, the study was limited by medical records being examined, in retrospect after 5 and 10 years of follow-up, resulting in shorter follow-up times and thereby less robust results.

## Conclusions

AT treatment of nmRCC implied significantly higher risks of LR and DMR compared with PN. To minimize the risks of LR and DMR, the results of this study suggest that PN is preferred over AT as primary treatment, supporting the EAU guidelines to recommended AT mainly to frail and/or comorbid patients.

## Data Availability

The National Swedish Kidney Cancer Register (NSKCR) is an ogoing register not available for open access.

## Appendix

The National Swedish Kidney Cancer Register Steering Committee has the following members: Anca Dragomir, anca.dragomir@akademiska.se; Anders Kjellman, anders.kjellman@regionstockholm.se; Bianca Scholtz, Bianca.Scholtz@skane.se; Börje Ljungberg, borje.ljungberg@umu.se; Britt-Inger Dahlin, brittinger.dahlin@regionvasterbotten.se; Emma Mangelus, emma.mangelus@vgregion.se; Emma Ulvskog, emma.ulvskog@regionorebrolan.se; Linn Pettersson, linn.pettersson@rjl.se; Magnus Lindskog, magnus.lindskog@regionstockholm.se; Marcus Thomasson, marcus.thomasson@onkologi.umu.se; Martin Johansson, martin.johansson@med.lu.se; Mikael Hellström, mikael.hellstrom@xray.gu.se; Per Lindblad, per.lindblad@oru.se, Per Skoglund, per.skoglund@regionostergotland.se; Pernilla Sundqvist, pernilla.sundqvist@regionorebrolan.se; Sigrid Isaksson, Sigrid.Isaksson@skane.se; Stephanie Bonn, stephanie.bonn@ki.se; Susanna Holst, susanna.holst@skane.se; Sven Lundstam, sven.lundstam@vgregion.se; Ulrika Harmenberg, ulrika.harmenberg@regionstockholm.se.

## Abbreviations

ACM: All-cause mortality
AT: Ablation therapy
ccRCC: Clear cell RCC
CI: Confidence interval
DMR: Distant metastatic recurrence
GEE: Generalized estimated equations
HR: Hazard ratio
LR: Local recurrence
nmRCC: Nonmetastatic RCC
NSKCR: National Swedish Kidney Cancer Register
PN: Partial nephrectomy
RCC: Renal cell carcinoma
SD: Standard deviation
SE: Standard error
